# Gelatin/carboxymethyl cellulose edible films: modification of physical properties by different hydrocolloids and application in beef preservation in combination with shallot waste powder

**DOI:** 10.1039/d3ra00430a

**Published:** 2023-03-29

**Authors:** Thi Tuong Vi Tran, Nhu-Ngoc Nguyen, Quoc-Duy Nguyen, Tran-Phong Nguyen, Tuyet-Ngan Lien

**Affiliations:** a Faculty of Environmental and Food Engineering, Nguyen Tat Thanh University Ho Chi Minh City 754000 Vietnam nqduy@ntt.edu.vn

## Abstract

In this work, a gelatin/carboxymethyl cellulose (CMC) base formulation was first modified by using different hydrocolloids like oxidized starch (1404), hydroxypropyl starch (1440), locust bean gum, xanthan gum, and guar gum. The properties of modified films were characterized using SEM, FT-IR, XRD and TGA-DSC before selecting of best-modified film for further development with shallot waste powder. SEM images showed that the rough or heterogeneous surface of the base was changed to more even and smooth depending on the hydrocolloids used while FTIR results demonstrated that a new NCO functional group non-existent in the base formulation was found for most of the modified films, implying that the modification led to the formation of this functional group. Compared to other hydrocolloids, the addition of guar gum into the gelatin/CMC base has improved its properties such as better color appearance, higher stability, and less weight loss during thermal degradation, and had minimal effect on the structure of resulting films. Subsequently, the incorporation of spray-dried shallot peel powder into gelatin/CMC/guar gum was conducted to investigate the applicability of edible films in the preservation of raw beef. Antibacterial activity assays revealed that the films can inhibit and kill both Gram-positive and Gram-negative bacteria as well as fungi. It is noteworthy that the addition of 0.5% shallot powder not only effectively decelerated the microbial growth but also destroyed *E. coli* during 11 days of storage (2.8 log CFU g^−1^) and the bacterial count was even lower than that of uncoated raw beef on day 0 (3.3 log CFU g^−1^).

## Abstract 2

In this work, a gelatin/carboxymethyl cellulose (CMC) base formulation was first modified by using different hydrocolloids like oxidized starch (1404), hydroxypropyl starch (1440), locust bean gum, xanthan gum, and guar gum.

## Graphical abstract

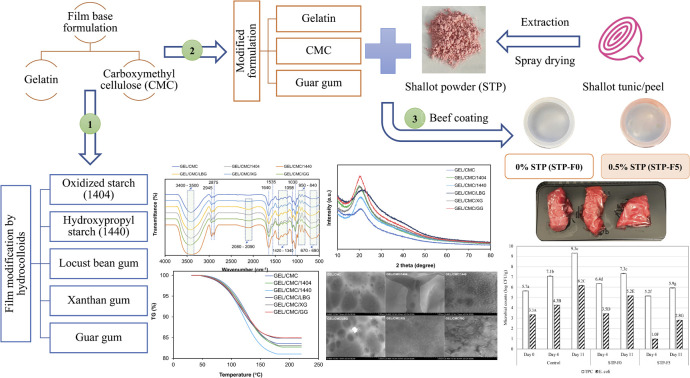

## Introduction

Environmental pollution is a significant issue that the world has been dealing with; in this context, non-biodegradable packaging forms are a leading concern due to the pressure to tackle the increasing amount of packaging trash globally.^1^ Currently, researchers are working hard to effectively reduce, reuse, recycle and recover packaging materials.^2^ In addition, biodegradable and edible packaging composed of biodegradable polymers consisting mostly of proteins and polysaccharides with combinations of hydrocolloids (such as chitosan, carboxymethyl cellulose, and starch) are gaining interest.^3^ Researchers and food corporations are interested in smart packaging, a new generation of food packaging, owing to its potential to improve food quality, hygiene and safety while minimizing environmental problems through the use of natural preservatives.^4,5^ A convenient packaging system based on the incorporation of active compounds (antimicrobials and/or antioxidants) or intelligent indicators in the food packaging material to control and limit undesirable changes of food quality is required.^6,7^ Intelligent compounds offer information on the quality of packaged food, while active compounds increase or maintain the quality of the product.^8^

The term “active packaging” refers to a kind of packaging in which the product, the packaging, and the surrounding environment all work together for the benefit of the product, such as shelf-life extension of accomplishing desired attributes through its effects on physiological, chemical, physical and microbiological changes due to microbial action or infection by insects.^9^ The active packaging system utilizes substances such as oxygen scavengers, carbon dioxide generators, odor absorbers, relative humidity regulators, antibacterials, and antioxidants. In addition, intelligent packaging may be described as a packaging system able to conduct intelligent tasks such as detecting, recording, tracing, and notifying product safety in order to offer information for making decisions about the shelf life of products.^10^ Polysaccharides and proteins are polymers commonly used as film materials due to their ability to gel in aqueous solutions and their high hydrophilicity to prevent the permeation of atmospheric oxygen.^11^ However, these edible films often have lower mechanical strength (tensile strength) than plastic-based films and have poor resistance to water vapor due to their hydrophilic nature.^12^

Gelatin is a protein obtained mostly from pig and cow skin, fish, and animal bones; hence, it is regarded as a renewable biodegradable material with the potential to be used in the development of preservation films with good gas permeability and high mechanical strength.^13^ In addition, gelatin films have a comparatively high capacity for water absorption owing to their high inherent hydrophilicity.^14^ Due to its cheap cost, availability, and good film-forming capabilities, carboxymethyl cellulose (CMC) is one of the oldest natural polysaccharides used widely in packaging. However, CMC-based films' weak mechanical characteristics and susceptibility to moisture have severely restricted their use.^15^ As a solution, mixing gelatin with biofilm forming agents such as CMC is a promising method to alleviate the disadvantages of gelatin films, which provide a good barrier of lipids, oxygen, and carbon dioxide.^16^ In fact, there are several methods used to modify the physical and mechanical properties of edible coatings including chemical (chemical addition), physical (ultrasonic treatment), and enzymatic (transglutaminase) treatment. Regarding the two latter methods, ultrasonic treatment led to slightly reduced water vapor permeability, increased tensile and puncture strengths while the addition of transglutaminase only increased puncture deformation compared with the control whey protein-based films.^17^ Transglutaminase is a cross-linked enzyme that catalyses the formation of isopeptide bonds between protein molecules, so it has potential applications to improve physicochemical properties such as solubility, water holding capacity, and emulsifying ability of protein-based edible films or coatings.^18^ Among these, the chemical method is considered a popular method due to the variety of additives including hydrocolloids (xanthan gum,^15^ oxidized guar gum,^19^ tara gum/locust bean gum,^20^ and gum tragacanth/locust bean gum^21^) as copolymers or alginate dialdehyde^22^ and pullulan^23^ as cross-linking agents. There are many studies using different hydrocolloids to modify the structure and mechanical properties of gelatin-based films; for example, gelatin-based films modified by CMC/xanthan gum has been improved in terms of water vapor permeability, UV blocking, thermal stability and tensile strength.^15^ Additionally, oxidized guar gum reduced solubility, water vapor permeability but increased tensile strength by two times^19^ while tara gum/locust bean gum^20^ and gum tragacanth/locust bean gum^21^ produced more elastic films with better mechanical properties. However, little research has been conducted on the utilization of modified starches.

Meat as a post-mortem edible ingredient derived from a live animal containing approximately 70% water, 20% protein, lipid and other constituents.^24^ Due to its high nutritional value, meat is very susceptible to physical, chemical and microbiological deterioration during which significant changes occur, including protein oxidation, lipid oxidation and increase in metmyoglobin.^25^ The spoilage and contamination of food by microorganisms is one of the major problems and no radical control method has been found.^26^ There have also been numerous investigations into the use of gelatin-based edible films in combination with various plant extracts to extend the shelf-life of meat and meat products, such as gelatin/κ-carrageenan/zein double layer films containing curcumin for preservation of grass carp fillets,^27^ gelatin/CMC/chitin nanofiber with *Trachyspermum ammi* essential oil for preservation of raw beef,^28^ and gelatin–chitosan films containing *Ferulago angulate* essential oil for preservation of turkey meat.^29^ Red onions or shallots (*Allium ascalonicum* L.) are the popular vegetable that contains many nutrients, including proteins, crude fibers, a wide range of vitamins,^30^ and flavonoids, particularly anthocyanin red pigments (10% of total flavonoids).^31–33^ Shallots show notable antibacterial on account of sulfur-containing chemicals (allyl trisulfide, allyl-cysteine, and diallyl sulfide) and flavonoids (quercetins, flavones, and anthocyanins), the two primary sources of antioxidant and antibacterial activity in this plant.^34,35^ Anthocyanins, which are naturally occurring pigments and present in many colored fruits and vegetables, have been considered a useful pH indicator in smart food packaging because of their color shift at various pH levels.^36^ Therefore, the application of edible coatings containing active ingredients from shallots shows high applicability in preserving fresh meat products.

This study aims to compare the microstructure of gelatin/CMC-based edible films fortified with a variety of hydrocolloids, such as gums (xanthan gum, guar gum, and locust bean gum) and modified starches (hydroxypropyl and oxidized starches), and to assess the feasibility of incorporating shallot waste into modified gelatin/CMC-based films for meat preservation. The shallot preparation of our interest was spray-dried powder because of its convenience in storage and transportation.

## Experimental

### Materials and chemicals

Raw beef tenderloin and shallot wastes were collected from a local market in Ho Chi Minh city (Vietnam). The hydrocolloids used in the study include gelatin with 250 Bloom (Weishardt International); Aqualon™ carboxymethyl cellulose (Ashland) with molecular weight of 725 000, viscosity of 1500–3000 mPa s at 1% solution; Ziboxan® PM200 xanthan gum (Deosen Biochemical (Ordos) Ltd) with a viscosity of 1200–1600 cp at 1% solution; guar gum (Shree Ram India Gums Pvt. Ltd) with a viscosity of 5000 cp at 1% solution; CESAGUM® locust bean gum (Tate & Lyle PLC); OS17 oxidized starch E1404 (Golinse) with a viscosity of 2200–2800 cp at 11.9% solution, CLEAGUM® CK2020 hydroxypropyl starch E1440 (Roquette); and maltodextrin (Baolingbao Biology Co., Ltd) with DE 8–10, solubility >98%.

Pathogenic microorganisms, including seven Gram-negative bacteria (*Shigella sonnei* ATCC 9290, *Escherichia coli* ATCC 8739, *Citrobacter freundii* ATCC 8090, *Salmonella typhi* ATCC 6539, *Vibrio parahaemolyticus* ATCC 17802, *Proteus mirabilis* ATCC 25933, *Campylobacter jejuni* ATCC 33291), three Gram-positive bacteria (*Staphylococcus aureus* ATCC 6538, *Bacillus cereus* ATCC 11778, *Listeria monocytogenes* ATCC 13932), and one yeast strain (*Candida albicans* ATCC 10231) were kept frozen in Mueller–Hinton Broth (MHB) medium containing 15% v/v glycerol.

Folin–Ciocalteu reagent, gallic acid, 2,2-diphenyl-1-picrylhydrazyl (DPPH), 2,4,6-Tris(2-pyridyl)-*s*-triazine (TPTZ), 2,2′-azino-bis(3-ethylbenzothiazoline-6-sulfonic acid) (ABTS), and Trolox were purchased from Sigma-Aldrich (Singapore). Mueller–Hinton Agar (MHA), Mueller–Hinton Broth (MHB), Plate Count Agar (PCA), Tryptone Bile X-glucuronide agar (TBX) were purchased from Hi-Media Laboratory (Mumbai, India). Other chemicals were of analytical grade.

### Modification of gelatin/CMC-based films by the fortification of different hydrocolloids

To prepare gelatin/CMC films, 1 g of gelatin was soaked in 95 mL of distilled water for 15 min and heated to 60 °C in a thermostatic bath WB–22 (Daihan Scientific, Korea). Upon heating, the mixture of 0.5 g CMC and 0.5 g of each of different gums and modified starches, namely oxidized starch (1404), hydroxypropyl starch (1440), locust bean gum (LBG), xanthan gum (XG), and guar gum (GG) was gradually added to the gelatin solution, continuously stirred, and then allowed to cool. Subsequently, glycerol (2 g) was added and the resulting solution was further sonicated (40 kHz, 150 W) in a GT-1860QTS ultrasonic cleaner (GT Sonic, China) for 10 min at 50 °C for foam removal, followed by pouring into a glass Petri dish of 90 mm diameter to reach 5 mm thickness of 30 mL polymer solution and dried at 60 °C for 5–6 h in the LO-FS100 forced convection oven (LK Lab, Korea). Eventually, the resulting films were stored in a desiccator at ambient temperature for 2 days for conditioning.

### Incorporation of shallot tunic powder into gelatin/CMC-based films

After the gelatin/CMC based films were modified by the addition of different hydrocolloids, one combination was selected to evaluate the applicability of incorporation of plant-derived antimicrobials (shallot waste). To prepare the shallot tunic extracts (STE), 10 g of ground dried shallot tunic (moisture content of 12.51 ± 0.24%) was extracted with 300 mL of methanol : 1% HCl (volumetric ratio of 7 : 3) at 60 °C for 30 min. After extraction, the extract was obtained by filtering through Whatman filter paper no. 2 and vacuum evaporated at 55 °C for 4 h using a Hei-VAP value rotary evaporator (Heidolph Instruments, Germany). The concentrate with soluble solid content of 5 °Brix was refrigerated at 4 °C for further analysis of physico-chemical properties (phenolic and anthocyanin contents), antioxidant activities and antibacterial activities. Subsequently, the concentrate was mixed with maltodextrin as a carrier for soluble solid content of the feed to reach 15 °Brix. Spray drying was performed in an SD-06AG spray dryer (Lab Plant Ltd, UK) with the feed flow rate and inlet temperature being fixed at 485 mL h^−1^ and 160 °C, respectively. The shallot tunic powder (STP) was stored in sealed polyethylene bags under freezing conditions for the production of edible films and analyzed for some selected properties similar to the extract. The STP-enriched gelatin/CMC-based films were prepared according to the procedure described above with the addition of 0.5 g STP after glycerol, which was referred to as STP-F5 film. Films without STP were considered as reference (STP-F0).

### Characterization of shallot tunic extracts (STE) and shallot tunic powder (STP)

#### Total phenolic content

The total phenolic content was performed according to the Folin–Ciocalteu method described according to ISO 14502-1:2005.^37^ The phenolic content was calculated based on the gallic acid standard curve and expressed in mg gallic acid equivalent per L (mg GAE per L) for STE and mg gallic acid equivalent per g on the dry weight (mg GAE per g DW) for STP.

#### Total monomeric anthocyanin content

The total anthocyanin content was measured through a differential pH method based on the characterization of anthocyanins pigments that change color at pH 1.0 and pH 4.5.^38^ The total monomeric anthocyanin content was expressed in mg cyanidin equivalent 3-glucoside per L (mg C3G per L) for STE and mg of cyanidin equivalent 3-glucoside per L on the dry weight (mg C3G per g DW) for STP.

#### DPPH free radical scavenging activity

Antioxidant activity was evaluated through DPPH free radical scavenging capacity based on the purple color change of DPPH solution (0.6 mM).^39^ The DPPH scavenging activity was calculated against the Trolox calibration curve and expressed in mg Trolox equivalent per L (mg TE per L) for STE and mg Trolox equivalent per g on the dry weight (mg TE per g DW) for STP.

#### ABTS cation radical scavenging activity

ABTS free radical scavenging activity was carried out based on the discoloration of ABTS (7.4 mM).^40^ The ABTS cationic radical scavenging activity was calculated against the Trolox calibration curve and expressed in mg Trolox equivalent per L (mg TE per L) for STE and mg Trolox equivalent per g on the dry weight (mg TE per g DW) for STP.

#### Ferric reducing antioxidant power (FRAP)

Ferric reducing antioxidant power (FRAP) was determined based on the chromophores formed between the working reagents (mixture of 0.3 M acetate buffer at pH 3.6, 0.01 M TPTZ prepared in 0.04 M HCl, and 0.02 M FeCl_3_·6H_2_O solution in a volumetric ratio of 10 : 1 : 1) with antioxidants.^41^ Ferric reducing antioxidant activity was calculated against the Trolox calibration curve and expressed in mg Trolox equivalent per L (mg TE per L) for STE and mg Trolox equivalent per g on the dry weight (mg TE per g DW) for STP.

#### Antibacterial activities – MIC and MBC

To determine minimum inhibitory concentration (MIC) of STE and STP, two-fold serial dilution was performed using 0.9% NaCl solution in a 96-well plate before adding MHB medium (50 μL) and bacteria cultures (50 μL) at a concentration of 10^8^ CFU mL^−1^ to reach total volume of 200 μL. After aerobic incubation (37 °C, 24 h), the MIC was verified as the concentration with no turbidity as indicative of microbial growth being observed. To determine minimum bactericidal concentration (MBC) of STE and STP, aliquots (20 μL) from wells with no detectable bacterial growth were plate poured on MHA plates, prior to incubation (37 °C, 18 h). The MBC was determined as the concentration at which no colonies were observed on the agar surface.

### Characterization of STP-enriched gelatin/CMC-based edible films (STP-F)

#### Moisture content

Moisture content (%) was determined after dehydrating films at 90 °C for 24 h to constant weight using the formula: (*W*_o_ − *W*_1_) × 100/*W*_o_; where, *W*_o_ and *W*_1_ are the sample weight before and after drying.

#### Film thickness and color attributes

Film thickness and the color attributes (CIELAB color space) were measured using a DM3025 IP54 digital micrometer (DML, UK) and NR110 precision colorimeter (3NH Technology Co. Ltd, China) at five random points on the film surface.

#### Film solubility in water

Films were cut into 20 × 20 (mm) squares and immersed in 50 mL of water for 24 h under normal conditions. After soaking, the film was dried at 90 °C for 24 h to constant weight to calculate the remaining dry mass. The water solubility of the film (%) was determined by the formula: (*W*_do_ − *W*_d1_) × 100/*W*_do_; where, *W*_do_ and *W*_d1_ are the dry mass of the film before and after immersion in water.

#### Absorption spectra

A 45 × 10 (mm) rectangular piece of film was cut and adhered to the interior of the quartz cuvette. The absorption spectra between 400 and 800 nm were then recorded using the UV-9000 spectrometer (Metash, China).

#### Microstructure and thermal analysis

The surface morphology of the films was determined by scanning electron microscopy (SEM) using S-4800 field emission scanning electron microscopes (Hitachi, Japan) at a scanning voltage of 10 kV. The functional groups were analyzed by Fourier transform infrared (FT-IR) spectroscopy using the Frontier NIR/MIR system (PerkinElmer, USA). The crystal structure of the films was investigated by X-ray diffraction (XRD) using Empyrean Diffractometer (PANalytical, Netherlands) with CuKα radiation in the range of 2*θ* from 5–80° at 0.02° resolution. The thermal properties of the films were determined by the thermogravimetry (TG) and differential scanning calorimetry (DSC) using the Labsys Evo TG-DSC 1600 C system (Setaram Instrumentation, France) over a temperature range of 30–220 °C at a rate of 10 °C min^−1^.

#### Application of STP-enriched gelatin/CMC/guar gum films on the preservation of raw beef

After being rinsed and chilled in ice water for 15 min, raw beef was immersed in a 0.9% saline solution, followed by cutting into 10 g cubes of 45 × 10 × 10 (*L* × *W* × *H* in mm) and coating with STP-enriched gelatin/CMC-based films. Meat samples were stored in covered containers (4 °C, 11 days) and analyzed for total plate count and *Escherichia coli* densities after 0, 4, and 11 days.

#### Microbiological control of raw beef during preservation

The density of pathogenic microorganisms on samples was analyzed by plate count technique. Briefly, 1 mL of the diluent was poured into Plate Count Agar (PCA) and Tryptone Bile X-glucuronide agar (TBX) medium for determination of total plate count and β-glucuronidase-positive *Escherichia coli*, respectively. The samples were then incubated under different conditions as described in ISO 4833-1:2013 for total plate counts (72 h, 37 °C) and ISO 16649-2:2001 for *E. coli* counts (24 h, 44 °C). The number of microorganisms is expressed as the log number of colony forming units per g of sample (log CFU g^−1^).

#### Statistical analysis

All statistical techniques, including normality test, homoscedasticity of variances, one-way ANOVA, and *post hoc* Tukey test, were performed at 5% significance level by using R version 4.1.2.

## Results and discussion

### Characterization of gelatin/CMC films modified by different hydrocolloids

#### Moisture content, thickness, and color attributes

**Fig. 1:**
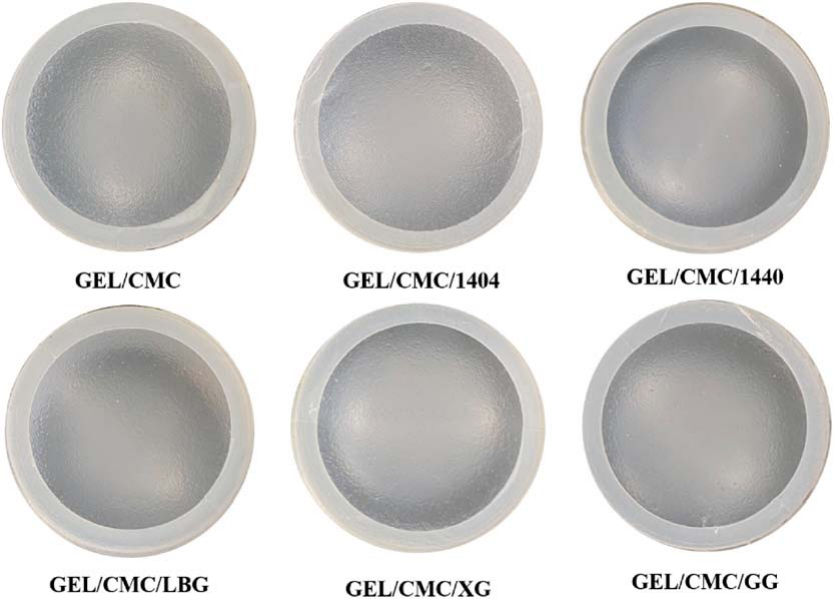
Visual appearance of gelatin/CMC-based films and their modification by the addition of different hydrocolloids (GEL/CMC/x).

The images, colors and thickness of gelatin/CMC-based films using different hydrocolloids are shown in Fig. 1 and Table 1. In general, the films have good visual appearance when observed with the naked eye with a smooth crack-free surface, demonstrating the film-forming ability of hydrocolloid mixture in terms of sensory perception. The addition of hydrocolloids to the gelatin/CMC film formulation reduced the moisture content of the resulting films with the moisture value of modified films being in the range of 24.85–27.65% which is higher than that of the control films (28.73%). This can be explained by the increased solid content in modified formulations.^15^ In addition, the thickness of the six films are similar and ranges from 0.13 to 0.14 mm. In terms of color attributes, the brightness of the GEL/CMC/1440 films was significantly reduced compared to the control film while the other films were almost identical. Hazirah *et al.*^15^ investigated the addition of xanthan gum to the gelatin/CMC formulation and concluded that the addition of xanthan gum resulted in a compact film network with gelatin and CMC through crosslinking. In addition, not only can xanthan gum interact with gelatin through hydrogen bonding and hinder –OH functional groups from interacting with water and reducing the moisture content of the gelatin film^15^ but also reduce its ability to bind water, thereby reducing the solubility of the film^19^ and darken the color of films.^15,42^

**Table 1:** Some selected physical properties of modified gelatin/CMC films (GEL/CMC/x) and gelatin/CMC/guar gum films fortified with 0.5% shallot tunic powder (STP-F5)^a^

	GEL/CMC	GEL/CMC/1404	GEL/CMC/1440	GEL/CMC/LBG	GEL/CMC/XG	GEL/CMC/GG (STP-F0)	STP-F5
Moisture (%)	28.73 (0.66)^a^	26.23 (0.57)^b^	27.65 (0.69)^a^	24.85 (0.56)^b^	25.63 (0.46)^b^	25.86 (0.35)^b^	25.54 (0.68)^b^
Thickness (mm)	0.13 (0.01)^ab^	0.13 (0.01)^ab^	0.13 (0.01)^ab^	0.14 (0.01)^b^	0.14 (0.01)^b^	0.13 (0.01)^ab^	0.12 (0.01)^a^
Solubility (%)	100%^a^	100%^a^	100%^a^	100%^a^	100%^a^	100%^a^	92.54 (1.21)^b^
L*	63.24 (1.18)a	64.38 (2.36)^a^	30.31 (1.35)^b^	46.77 (0.07)^c^	59.46 (2.96)^d^	55.02 (3.00)^d^	48.36 (0.16)^e^
a*	−1.41 (0.06)^a^	−0.85 (0.04)^b^	−0.41 (0.02)^c^	−0.13 (0.01)^d^	−1.22 (0.03)^e^	−1.57 (0.03)^f^	22.85 (0.40)^g^
b*	5.70 (0.25)^a^	8.79 (0.27)^b^	6.79 (0.16)^c^	3.40 (0.18)^d^	4.95 (0.23)^e^	5.82 (0.18)^a^	22.49 (0.11)^f^
C*	6.02 (0.32)^a^	8.80 (0.26)^b^	6.86 (0.19)^c^	3.41 (0.19)^d^	6.43 (0.17)^a^	6.00 (0.19)^a^	18.61 (0.06)^e^
*h*°	104.09 (2.57)^a^	95.37 (1.68)^b^	98.03 (2.49)^b^	93.81 (3.33)^b^	102.81 (2.97)^a^	104.35 (1.05)^a^	44.94 (1.67)^c^

a Notes: the results were presented as mean (standard deviation) of triplicates and different letters in the same columns indicate that the mean values were significantly different at 95% confidence level. Film formulations were modified by the addition of E1404 oxidized starch (GEL/CMC/1404), E1440 hydroxypropyl starch (GEL/CMC/1440), locust bean gum (GEL/CMC/LBG), xanthan gum (GEL/CMC/XG), and guar gum (GEL/CMC/GG).

#### SEM

**Fig. 2:**
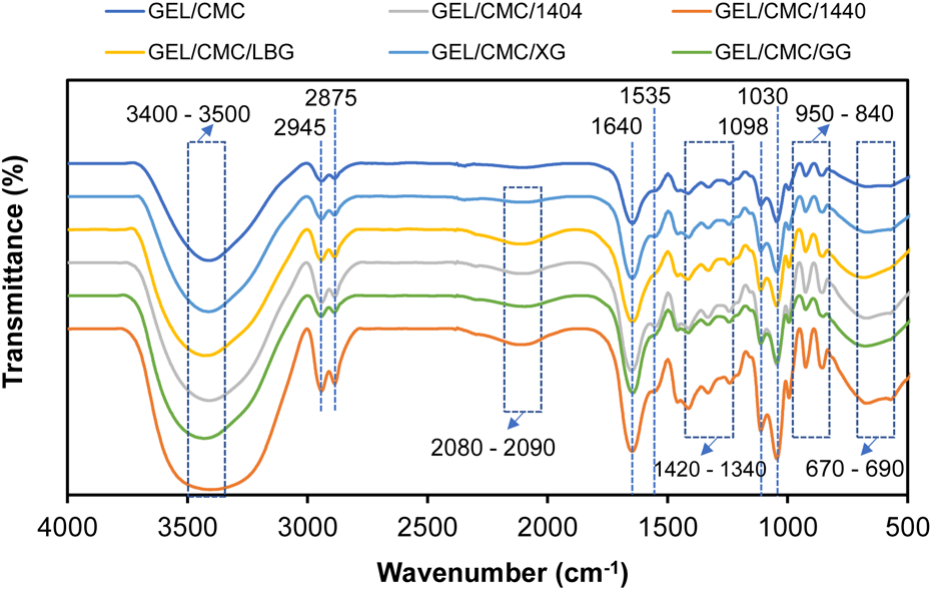
FTIR spectroscopy of gelatin/CMC-based films and their modification by the addition of different hydrocolloids (GEL/CMC/x).

The results of surface morphology are presented in Fig. 2. In general, under 200 00× magnification, several dark spots present on the surface of gelatin/CMC, as a result of the reorganization of protein chains in gelatin structure during the drying process forming a fibrous structure.^43,44^ GEL/CMC has a rough or heterogeneous surface. Its modification with 1404, 1440, LBG, XG and GG shows a slight change in the original surface structure of GEL/CMC. The modification of XG and 1440 has filled the uneven porous on the surface of GEL/CMC which leads to a significant reduction in the dark spot zone on GEL/CMC surface while the modification of 1404 and LBG show a slight effect on the surface of GEL/CMC. Nonetheless, the modification of GG heavily affects the GEL/CMC surface as its surface not only filled out the porous structure of base material but also created some cracks on the surface which leads the modified material surface becoming rough and uneven but in a different form with the original material.

#### FTIR

**Fig. 3:**
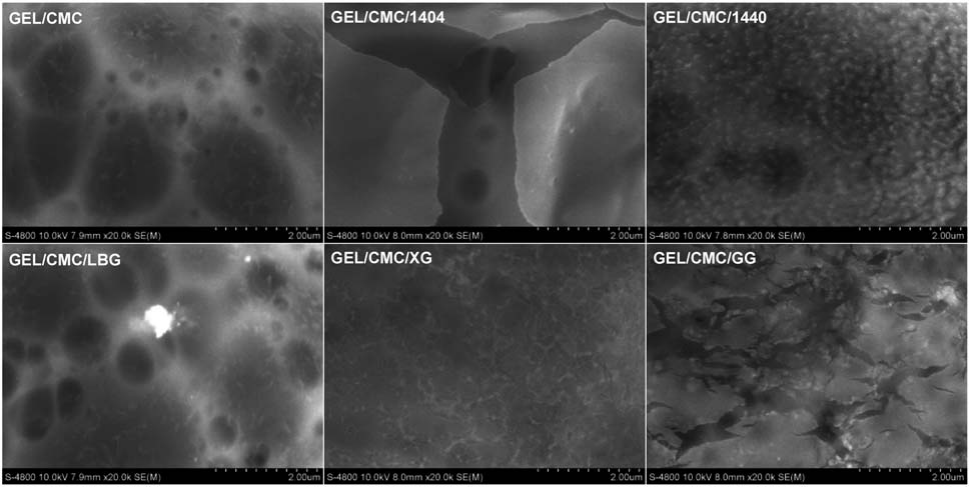
SEM images of gelatin/CMC-based films and their modification by the addition of different hydrocolloids (GEL/CMC/x).

The FTIR spectroscopy of different gelatin/CMC-based films as modified by different hydrocolloids in Fig. 3 shows that the GEL/CMC film shows the main functional group of –OH stretch vibration band at 3400–3500 cm^−1^. The reason for this could be explained by the interaction of intermolecular between hydroxyl group in CMC and carboxyl group in gelatin.^44,45^ The other stretch band was found as well at band 3000–2850 cm^−1^ representing for =C–H and CH_2_ group (amide-III). Vibration at band 1535–1640 cm^−1^ indicates the solid carboxylate group includes –C=O and –COO. The vibration at band 1420–1130 cm^−1^ indicates –CH bending. The vibration at band 1130–1000 cm^−1^ indicates the possibility of –CN or –NH. The vibration at the band 1000–840 cm^−1^ indicates a phenolic component and the vibration at band 840–500 cm^−1^ indicates the presence of –C–H in the aromatic ring. These are in good agreement with some studies on gelatin composite films.^45,46^ Moreover, the vibration at band 2300–2000 cm^−1^ indicates the stretching of the NCO group, which is not found on GEL/CMC which means that the modification leads to the formation of this NCO group.^47^ CMC in interaction with gelatin causes a conformational change of the gelatin chain, reducing the presence of single helices and disordered structures as well as increasing antisymmetric and symmetric vibrations of C=O and C–O bonds due to the breakdown of hydrogen bonds formed between polypeptides chains.^46,48,49^

#### XRD

**Fig. 4:**
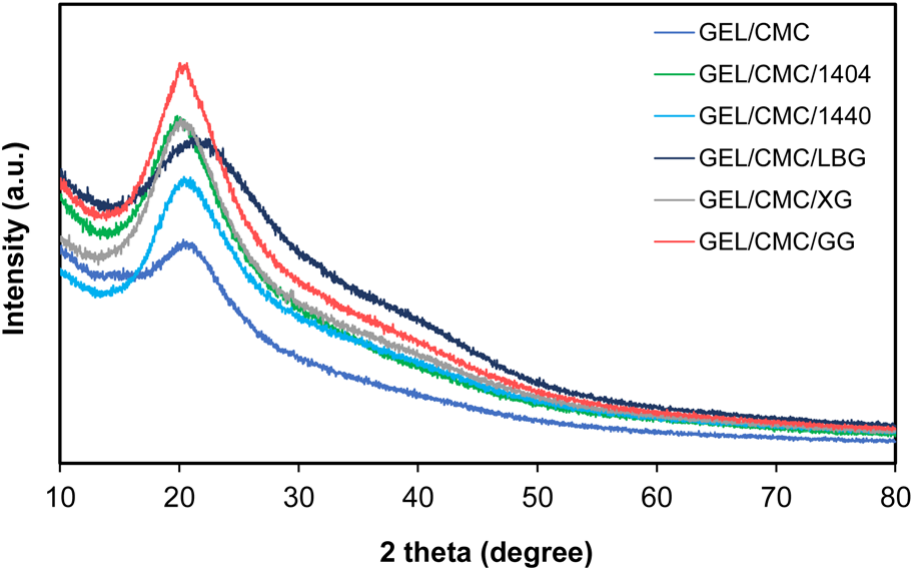
X-ray diffraction of gelatin/CMC-based films and their modification by the addition of different hydrocolloids (GEL/CMC/x).

The crystalline of GEL/CMC and GEL/CMC/x is demonstrated in Fig. 4. It is obvious that there was one main peak at 2*θ* of 20° on GEL/CMC indicating the amorphous structure of the material. The modification with 1404, 1440, XG and GG created no new peak but increased the intensity of the crystalline structure on the GEL/CMC. This could be explained by the aggregation of more molecules from the agents that have been substituted and branched during the modification process on GEL/CMC, respectively. Especially, by modification with GG, the highest intensity of the crystalline on the sample was found.^20^ Interestingly, the modification with LBG resulted in the peak of crystalline shift to 2*θ* of 21.5° instead of 2*θ* of 20°. The reason causing the shift in the sample is that mannose and galactose in LBG have been converted into uronic acids which affect polysaccharide chain formation and the way they were presented in the phase of solid.^50^ The amorphous structure of the films shown by the peak at 2*θ* = 20–21.5° could be the result of the double-helical configuration and the amorphous solid-state nature of CMC, leading to higher water vapor permeability.^15,51^ Good water vapor permeability also helps to limit the condensation of water vapor on the surface of the food and is suitable for use as a primary package.^51^

#### TGA-DSC

**Fig. 5:**
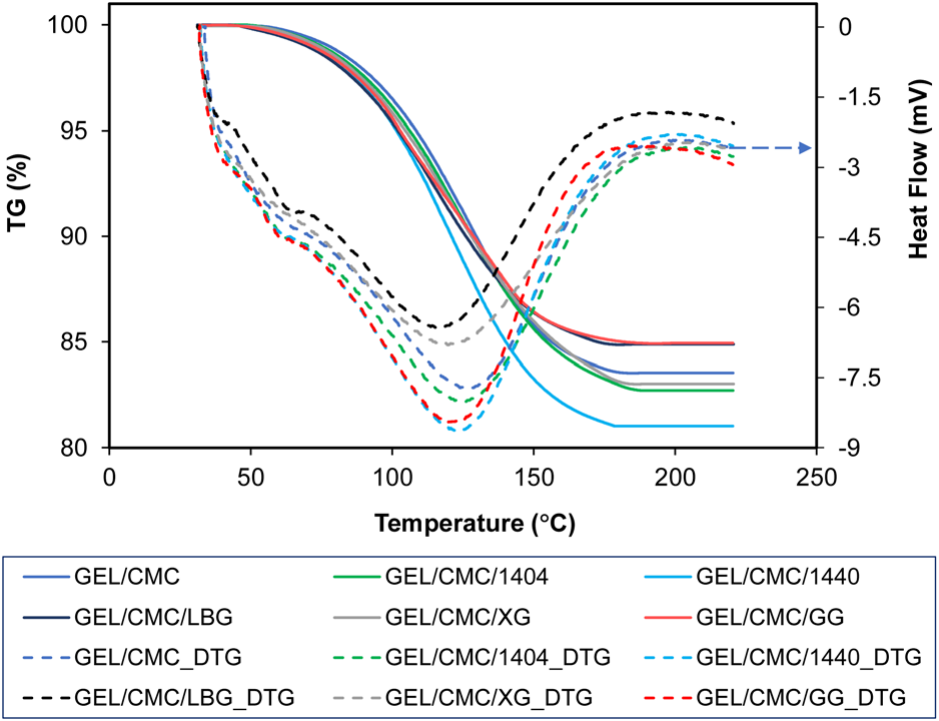
Thermal degradation of gelatin/CMC-based films and their modification by the addition of different hydrocolloids (GEL/CMC/x).

Thermal degradation behavior of the film and its modification monitored by TGA-DSC is shown in Fig. 5 and the data of onset temperature, peak temperature and end temperature are shown in Table 2. From the thermal degradation chart and table, it is shown that all samples have different onset temperature, peak temperature, end temperature and weight loss. In detail, the degradation of GEL/CMC sample started at 53 °C, reached the peak temperature at 126.7 °C, and completed at 184 °C with a weight loss of 16.41%. Upon modification, the degradation of GEL/CMC/XG started at 45 °C, reached the peak temperature at 122.5 °C and end at higher temperature of 188 °C with the weight loss of 16.93%, whereas the degradation of GEL/CMC/1404 had the same onset temperature with GEL/CMC/XG at 45 °C, reached the peak temperature at 124.5 °C and completed at the highest temperature of 190 °C with a slightly higher weight loss of 17.03%. The sample which had the highest weight loss of 18.93% was GEL/CMC/1440 at end temperature of 181.8 °C when the degradation was completed while the onset temperature at 46.5 °C and the peak temperature at 122.4 °C. In the meantime, the GEL/CMC/LBG demonstrated the thermal degradation process from starting to ending all at the lowest temperature, namely onset temperature at 40 °C, peak temperature at 116 °C and end temperature of 180 °C with the lowest weight loss of 15.09%. Interestingly, the GEL/CMC/GG had the same weight loss as GEL/CMC/LBG but with a higher end temperature of 188 °C while an onset temperature at 44 °C and the peak temperature at 122 °C.

**Table 2:** Thermal degradation properties of gelatin/CMC-based films and their modification by the addition of different hydrocolloids (GEL/CMC/x)

Sample	Temperature (°C)			Δ*m* (%)
	Onset	Peak	End	
GEL/CMC	53.00	126.70	184.00	16.41
GEL/CMC/1404	45.00	124.50	190.00	17.03
GEL/CMC/1440	46.50	122.40	181.80	18.93
GEL/CMC/LBG	40.00	116.00	180.00	15.09
GEL/CMC/XG	48.00	122.50	188.00	16.93
GEL/CMC/GG	44.00	122.00	188.00	15.14

The thermal degradation profiles range from 40 to 190 °C in the chart indicates that the weight loss was mainly the evaporation of moisture and unstable/decomposed components of the investigated film samples, respectively.^52,53^ Furthermore, the weight loss of film during thermal degradation is due to the degradation of protein-based amino acids which are the major components of gelatin-based films, and the carbonyl group of the CMC side chains for CMC-based films.^44,54,55^ On the other hand, the crystalline structure of the hydrocolloids added to the membrane formulation is thought to increase the thermal stability, which is attributed to the intermolecular and intramolecular interactions of the macromolecules.^44^

Based on the results from SEM, FTIR, XRD, and TGA-DSC, the modification of GG to GEL/CMC demonstrated the best properties compare to other modification agents such as better color appearance, fewer changes to the structure of GEL/CMC, functional groups were comparable, higher stability in thermal degradation and less weight loss. Hence, GEL/CMC modified by GG has been selected as a new base film for further addition of shallot tunic extract in order to synthesize the edible films for beef preservation.

### Characterization of shallot tunic extracts (STE) and spray-dried powder (STP)

**Fig. 6:**
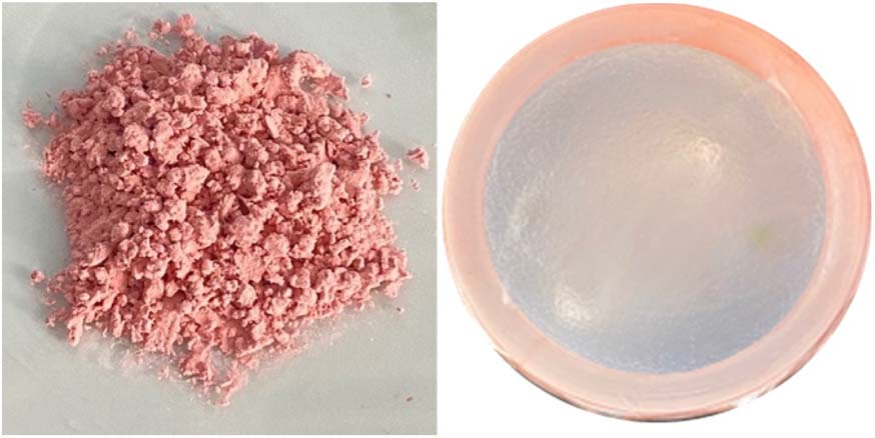
Visual appearance of shallot tunic powder (left) and gelatin/CMC/guar gum films after the addition of shallot tunic powder at 0.5% (right).

**Fig. 7:**
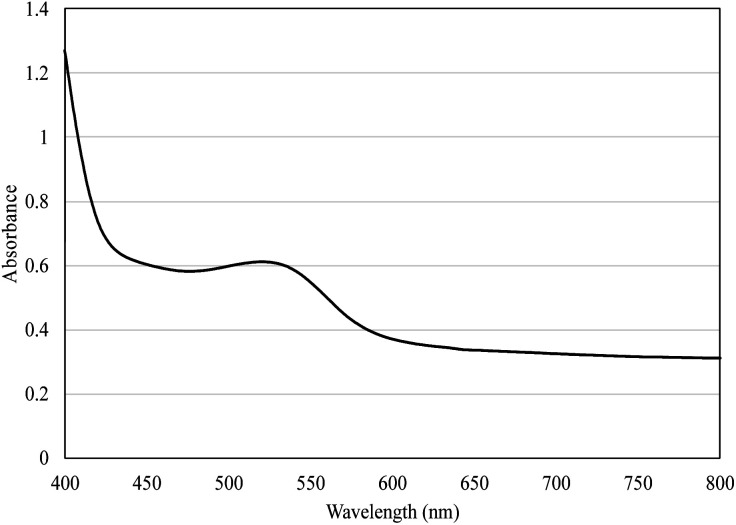
Absorption spectra of gelatin/CMC/guar gum films enriched with 0.5% shallot tunic powder (STP-F5) in the range of 400–800 nm.

The high anthocyanin content present in the form of red cationic flavylium ions when extracted with acidic solvents^56^ in STE (69.72 mg C3G per L) and STP (1.44 mg C3G per g DW) is responsible for the characteristic red color of STP and STP-F5 film (Fig. 6) with the maximum absorption at 520 nm (Fig. 7). It is noteworthy that the maltodextrin-assisted spray drying exerted positive influence on the retention of anthocyanins and antioxidant activity of spray-dried shallot (Table 3), as reported in various studies on the microencapsulation of anthocyanins.^57–59^ Shallot powder shows potential application for incorporation into edible films for food preservation due to its remarkably high phenolic content and antioxidant activity.

**Table 3:** Some selected physicochemical properties of shallot tunic extract (STE) and shallot tunic powder (STP)^a^

	STP	STE
Moisture content (%)	4.89 ± 2.51%	—
Phenolic	51.58 ± 1.53 (mg GAE per g DW)	2613.54 ± 28.96 (mg GAE per L)
Anthocyanin	1.44 ± 0.10 (mg C3G per g DW)	69.72 ± 0.72 (mg C3G per L)
DPPH	36.67 ± 1.69 (mg TE per g DW)	1406.71 ± 36.85 (mg TE per L)
ATBS	30.44 ± 0.10 (mg TE per g DW)	1208.05 ± 22.71 (mg TE per L)
FRAP	64.11 ± 0.51 (mg TE per g DW)	1523.90 ± 41.11 (mg TE per L)
L*	58.92 ± 1.62	8.55 ± 0.23
a*	21.12 ± 0.60	26.40 ± 0.81
b*	13.72 ± 0.20	2.90 ± 0.03
C*	24.80 ± 0.54	26.62 ± 1.01
*h*°	33.85 ± 0.61	10.01 ± 0.40

a Notes: the results were presented as mean ± standard deviation of triplicates.

MIC (minimum inhibitory concentration) and MBC (minimum bactericidal concentration) are the minimum concentrations to inhibit and kill pathogenic microorganisms.^60^Table 4 shows the MIC and MBC values of STE and STP against 11 pathogenic microorganisms in which STE has the ability to inhibit and kill Gram-positive and Gram-negative bacteria, especially *Citrobacter freundii*, *Proteus mirabilis* and *Listeria monocytogenes* as well as fungi. This result is consistent with the studies on chemical composition and antibacterial ability of extracts obtained from onion flesh and skin.^61,62^ Sulfur-containing chemicals (allyl trisulfide, allyl-cysteine, and diallyl sulfide) and flavonoids (quercetins, flavones, and anthocyanins) are the two primary sources of antioxidant and antibacterial activity in this plant.^34,35^ However, shallot spray-dried powder was not capable of inhibiting *Bacillus cereus* at concentrations ≤4 g L^−1^ despite inherent inhibitory ability of extracts. This may be due to the heat loss of antibacterials during high-temperature spray drying.

**Table 4:** MIC (minimum inhibitory concentration) and MBC (minimum bactericidal concentration) of shallot tunic extract (STE) and shallot tunic powder (STP)

	STE^a^		STP^b^	
	MIC	MBC	MIC	MBC
*Shigella sonnei* ATCC 9290	4	2	1	4
*Escherichia coli* ATCC 8739	8	4	1	2
*Citrobacter freundii* ATCC 8090	16	8	1	4
*Salmonella typhi* ATCC 6539	4	2	1	2
*Vibrio parahaemolyticus* ATCC 17802	8	4	1	2
*Proteus mirabilis* ATCC 25933	16	8	1	4
*Campylobacter jejuni* ATCC 33291	4	2	1	2
*Staphylococcus aureus* ATCC 6538	4	2	1	2
*Bacillus cereus* ATCC 11778	4	2	n.d.	n.d.
*Listeria monocytogenes* ATCC 13932	16	8	1	2
*Candida albicans* ATCC 10231	4	2	0.5	1

a MIC/MBC are presented as the dilution factor of STE at 5 °Brix.

b MIC/MBC are presented as the concentration in g L^−1^. n.d. – not detected at ≤4 g L^−1^.

### Application of STP-enriched gelatin/CMC films in the preservation of raw beef

#### Characterization of STP-enriched gelatin/CMC films enriched with shallot tunic powder (STP-F)

Gelatin is one of the common ingredients used as edible coatings due to its film-forming properties and gelatin-based edible films are generally applied in meat to prevent moisture loss and lipid oxidation during cold storage.^63^Table 1 shows that STP-F5 film has the same moisture content and thickness as plain GEL/CMC/GG film with values of 25.54% and 0.12 mm, respectively. However, the solubility of the former (92.54%) was lower than the complete solubility of the original formulation. In addition, the decrease in lightness, the increase in a* value together with the hue of 44.94° showed that the resulting film from the addition of STP exhibits the characteristic red color of spray-dried powder shallot. Anthocyanin red pigments are mostly found in red onions and make up around 10% of the overall flavonoid content relative to the fresh weight.^31–33^

#### Microbiological qualities of raw beef coated by STP-F during storage

**Fig. 8:**
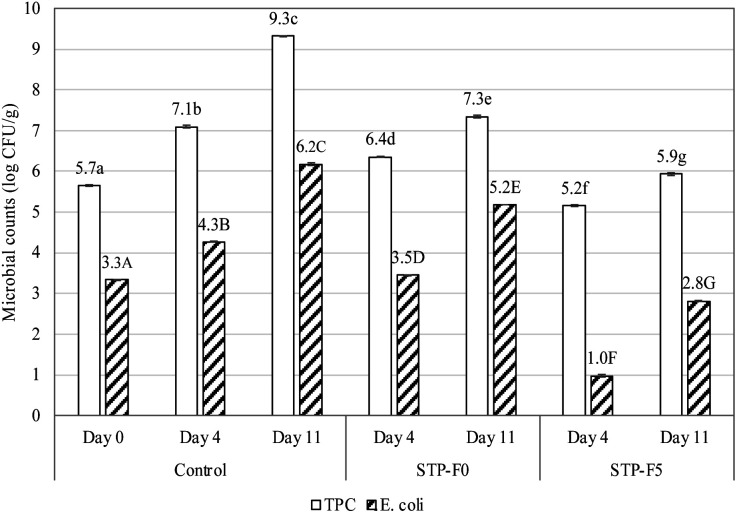
Changes in total plate count (TPC, log CFU g^−1^) and *E. coli* (log CFU g^−1^) of raw beef coated by gelatin/CMC/guar gum films enriched with 0% (STP-F0) and 5% (STP-F5) shallot tunic extracts during 14 days of storage in comparison with uncoated beef as control. Notes: different lowercase and uppercase letters indicate the significant difference (*p* < 0.05) among total plate count and *E. coli*, respectively.

Changes in total plate count and *E. coli* of raw beef coated by two edible films containing 0% (STP-F0) and 0.5% (STP-F5) shallot tunic powder during 11 days of storage in comparison with uncoated beef as control is shown in Fig. 8. The results showed that the microbial density of beef samples uncoated and coated with gelatin/CMC/guar gum films both increased during 11 days of storage. However, the microbial density of coated beef was consistently lower than that of control, even with the STP-F0 films which did not contain shallot powder. This may be explained by the fact that edible films can function as carriers that restrict microbial development,^64,65^ although to a lesser extent. Interestingly, despite the fact that gelatin and CMC lack antibacterial action, the gelatin/CMC combination has antibacterial activity against *S. aureus* and *E. coli.* This may be explained by the polyelectrolyte characteristics of β-(1 → 4)-glycosidic linkages when paired with gelatin capable of inhibiting bacteria.^44^

It is noteworthy that the *E. coli* densities of the STP-F5 coated beef samples on day 4 (1.0 log CFU g^−1^) and day 11 (2.8 log CFU g^−1^) were even lower than that of raw beef on day 0 (3.3 log CFU g^−1^). This demonstrates the effectiveness of shallot powder in destroying pathogenic microorganisms. This result is in accordance with the study of Irkin *et al.*^66^ on fresh beef soaked in onion extracts. Allicin, thiosulfinates, and their derivatives are responsible for the broad-spectrum action of *Allium* species against bacteria, fungi, viruses, and parasites, since they react with the sulfhydryl group of the functional proteins of these organisms.^67^

## Conclusions

Gelatin/CMC was used as a base for further modification with different hydrocolloids such as 1404, 1440, LBG, XG and GG. Most of the modified films showed a good visual appearance even observed by the naked eye. After the modification, the original rough and uneven structure of GEL/CMC was improved depending on the hydrocolloids used. Interestingly, FT-IR showed a newly formed functional group of NCO found on all modified GEL/CMC films. Particularly, modification with GG enhanced the thermal stability and therefore was chosen to incorporate with shallot spray-dried powder to develop active edible films for beef preservation based on its high antibacterial activity against 11 pathogenic microorganisms. The results revealed that the addition of shallot powder to the GEL/CMC/GG was efficient in prolonging the shelf-life of coated beef according to the destruction of *E. coli*.

## Author contributions

Thi Tuong Vi Tran: visualization; data curation; writing – original draft. Nhu-Ngoc Nguyen: investigation; visualization; data curation. Quoc-Duy Nguyen: conceptualization; data curation; investigation; visualization; methodology; writing – original draft; writing – review & editing. Tran-Phong Nguyen: investigation; data curation. Tuyet-Ngan Lien: investigation; data curation.

## Conflicts of interest

There are no conflicts to declare.

## Supplementary Material
